# Computer-Aided Diagnosis and Quantification of Cirrhotic Livers Based on Morphological Analysis and Machine Learning

**DOI:** 10.1155/2013/264809

**Published:** 2013-09-29

**Authors:** Yen-Wei Chen, Jie Luo, Chunhua Dong, Xianhua Han, Tomoko Tateyama, Akira Furukawa, Shuzo Kanasaki

**Affiliations:** ^1^College of Computer Science and Information Technology, Central South University of Forestry and Technology, Hunan, China; ^2^College of Information Science and Engineering, Ritsumeikan University, Shiga, Japan; ^3^Radiology Department, Tokyo Metropolitan University, Tokyo, Japan; ^4^Radiology Department, Shiga University of Medical Science, Shiga, Japan

## Abstract

It is widely known that morphological changes of the liver and the spleen occur during the clinical course of chronic liver diseases. In this paper, we proposed a morphological analysis method based on statistical shape models (SSMs) of the liver and spleen for computer-aided diagnosis and quantification of the chronic liver. We constructed not only the liver SSM but also the spleen SSM and a joint SSM of the liver and the spleen for a morphologic analysis of the cirrhotic liver in CT images. The effective modes are selected based on both its accumulation contribution rate and its correlation with doctor's opinions (stage labels). We then learn a mapping function between the selected mode and the stage of chronic liver. The mapping function was used for diagnosis and staging of chronic liver diseases.

## 1. Introduction

Chronic liver disease is a major worldwide health problem. Liver cirrhosis is a chronic liver disease that can be generally integrated into early, middle, and late stages. The appropriate treatment for liver cirrhosis depends strongly on the estimated stage. Since the late stage cirrhosis is often associated with an incidence of hepatocellular carcinoma, in radiology practice, early detection is essential for investigating the cause and slowing down the effects of cirrhosis [1]. Diagnosis and staging of chronic liver diseases are an important issue. The current clinical methods for detecting and staging cirrhosis are according to histological findings from results of liver biopsy or manually analyzing the morphological criteria on magnetic resonance (MR) imaging. However, liver biopsy subjects the patient to a risk of serious complications [2]. The manual analyzing process results in subjective diagnosis and is a difficult assignment for inexperienced radiologists. Consequently, researchers dedicated to develop computer-aided diagnosis (CAD) systems to assist the cirrhosis diagnosis. Liver tissue fibrosis is a distinctive characteristic of cirrhosis. Lesion tissue can be distinguished by having different texture in medical imaging. By far, all CAD schematics for cirrhosis diagnosis are exclusively based on texture analysis of the liver. Wang et al. [3] used the texture analyze with co-occurrence matrix method to analyze ultrasonograms of normal or diseased livers; although they proved that the texture analysis can help cirrhosis diagnosis, the diagnostic accuracy was not yet satisfied. The research group of Gifu University [4–6] and Kayaalti et al. [7] used texture features as input; they classified normal/cirrhotic liver by Artificial Neural Network (ANN) and Support Vector Machine (SVM), respectively. Both of them obtained high accuracy classification result. 

**Figure 1:**
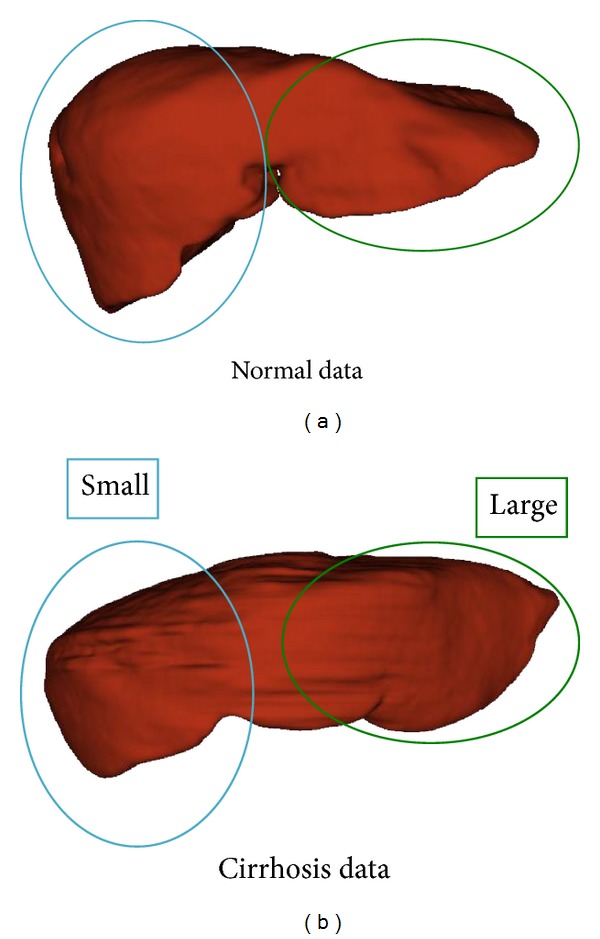
Typical shapes of normal (a) and cirrhosis (b) data.

However, texture analysis based methods have a major limitation that the texture difference between each cirrhosis stage is difficult to detect with current medical imaging techniques. As a result of this, it is practically impossible to estimate the proceeding stage of cirrhosis by texture analysis. Besides the tissue fibrosis, cirrhosis has another notable characteristic: morphological changes of the liver occur during the clinical course of chronic liver diseases [8]. The typical CT volumes are shown in Figure 1. The normal liver is shown in the left, and the cirrhotic liver is shown in the right. It can been seen that the cirrhotic liver will cause the left lobe's hypertrophy and the right lobe's atrophy. Though the morphologic change of the liver can be detected on computed tomography (CT), the visual assessment is subjective and limited in depicting minimal changes. 

The liver shape can be represented by a statistical shape model (SSM) [9, 10]. Compared with the conventional mathematical shape model such as a spherical harmonic model (SPHAM), SSM is statistically leaned from a population of objects or organs, and it is an object (or organ) specified shape model. The shape is constrained in its eigen subspace. To date little research has been done on the construction of statistical shape models of anatomical organs, such as brain [11], heart [12], liver [13], and spleen [14]. The SSM has also been applied to automatic segmentation of medical images [15–17]. In our previous works, we constructed a statistic shape model (SSM) of the liver and shown that coefficients of the model can be used for classification of cirrhotic livers and normal livers [18, 19]. The classification accuracy was about 60%–80%, which is depending on the number of training samples. In this paper, we improve our previous work from the following three aspects. (1) In order to improve the diagnosis accuracy of the cirrhotic liver, we newly constructed multiple SSMs (the liver SSM, the spleen SSM, and a joint SSM of the liver and the spleen) for morphological analysis, which is based on the well-known fact that the chronic liver diseases or cirrhosis will also cause significant morphological changes on spleen [20]. (2) The effective modes for diagnosis of the cirrhotic liver are selected based on both its accumulation contribution rate and its correlation with doctor's opinion (labels). In our previous work, we select the modes only based on its accumulation contribution rate. (3) In our previous work, we treated the diagnosis of the cirrhotic liver as a two-class (normal and abnormal) classification problem. It is not possible to estimate the proceeding stage of the cirrhotic liver. In this paper, we use support vector regression (SVR) [21] to learn a mapping function between the selected modes (mode coefficients) and the stage label. The mapping function is used to estimate the stage of the chronic liver diseases. 

This paper is organized as follows. In Section 2, we describe the construction of multiple statistical shape models (the liver SSM, the spleen SSM, and the joint SSM). In Section 3, we describe a mode selection method for effective mode selection. In Section 4, we briefly introduce SVR for the mapping function. Experimental results are presented in Section 5. The conclusion is given in Section 6.

## 2. Construction of Statistical Shape Models 

### 2.1. Preprocessing: Segmentation and Normalization

**Figure 2:**
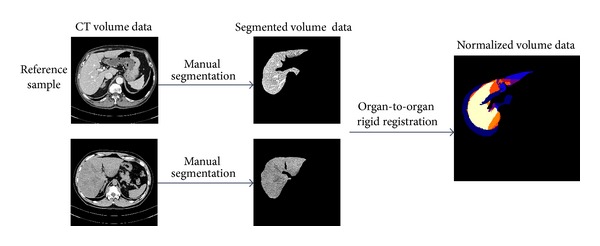
An example of preprocessing: segmentation and normalization.

As we mentioned in the previous section, the chronic liver diseases or cirrhosis will cause significant morphological changes on both liver and spleen. We constructed three statistical shape models: the liver SSM, the spleen SSM, and the joint SSM of the liver and the spleen. As the first preprocessing step, both the liver and the spleen are segmented manually in CT datasets. The segmentation is performed under the guidance of a physician in order to obtain accurate liver shape data and spleen shape data. Then, we randomly choose one sample as a reference sample and perform an organ-to-organ volume rigid registration as a data normalization step to remove the positional and rotational difference as much as possible. The example is shown in Figure 2. 

### 2.2. Statistical Shape Model Constructions

**Figure 3:**
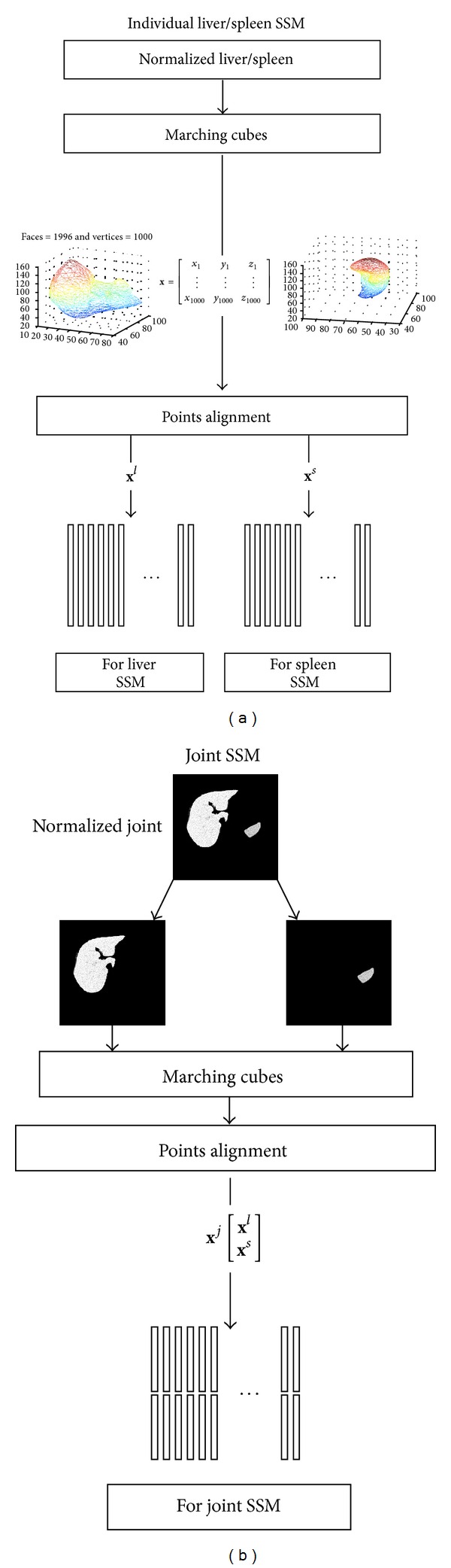
Flowcharts for construction of SSMs. (a) Individual liver/spleen SSM, (b) joint SSM of the liver and the spleen.

**Figure 4:**
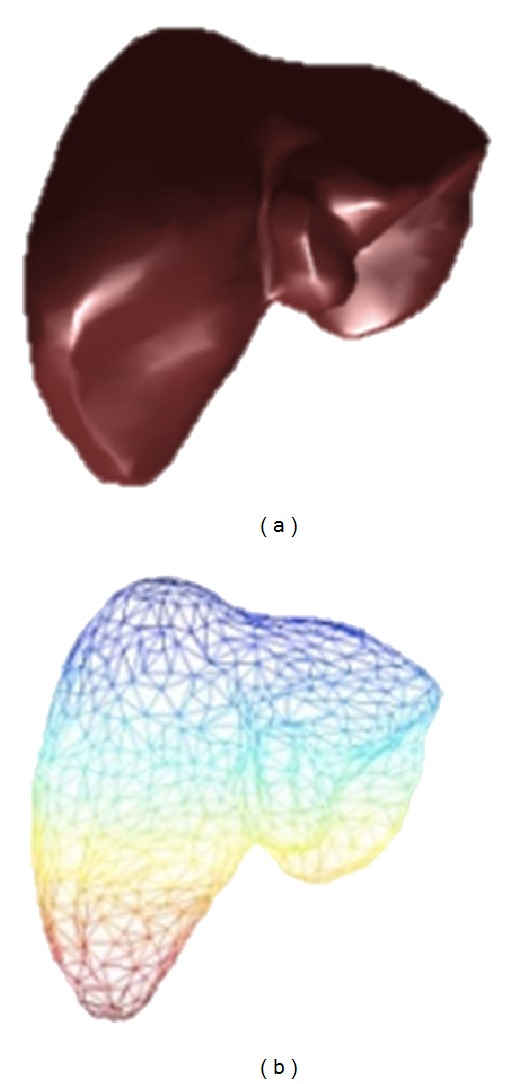
Marching cube method: (a) volume data. (b) Triangulated mesh surface data.

 The flowcharts for construction of the individual liver/spleen SSM and the joint SSM are shown in Figures 3(a) and 3(b), respectively. Each normalized organ volume (liver and spleen) is converted to a triangulated mesh surface by the use of marching cube algorithms [22]. Each surface contains 1000 vertex points as shown in Figure 4. Then, we use a nonrigid point matching method proposed by Chui and Rangarajan [23] to find the point correspondence between all of the datasets.

The liver shape or the spleen shape is represented as a shape vector **x** ^*l*^ or **x** ^*s*^ of three components corresponding to coordinates (*x*, *y*, *z*) of 1000 aligned vertex points that are obtained as the outputs of Marching cube algorithm and nonrigid point matching as shown in (1). For the joint SSM, the shape vector **x** is represented by [**x** ^*l*^, **x** ^*s*^]^*T*^. The dimensions for the individual organ shape vector and the joint organ shape vector are 3000 and 6000, respectively,

(1) xl or s=[x1,y1,z1,x2,y2,z2,…,x1000,y1000,z1000]T.

Assume *N* is the number of training samples. The mean shape **m** and covariance matrix **S** are calculated as

(2) m=1N∑i=1Nxi,S=1N∑i=1N(xi−m)(xi−m)T.

The modes of variation are found on the deviations of samples from the mean and are represented by *N* orthonormal eigenvectors (variation vectors) **v**_*j*_ of **S**, which are called as eigenshapes. The 3D shape of the liver can be represented as a linear combination of mean shape and eigenshapes as follows:

(3) x=m+∑jbjvj,

where *b*_*j*_ is the coefficient or weight of the *j*th mode of variation and is estimated by calculating **v** ^*T*^(**x** − **m**). It should be noted that the main variations could be captured by only a few top-leading modes (eigenvectors). The coefficients can be used as a feature vector of the 3D shape for image coding and quantitative analysis.

## 3. Selection of Effective Modes

 It is also an important issue to select effective modes, which control specific aspects of shape variations that are related to the morphological changes caused by cirrhosis. In addition to the conventional Accumulated Variance Contribution Rate (AVCR) based mode selection, we recently proposed a correlation based mode selection method and combine them to select the effective modes [24].

 In the correlation based mode selection, we are going to select modes which have strong correlation with doctor's opinions (labeled scores). Each sample data is labeled by doctors. The normal data is labeled as 0, and abnormal data is labeled as 1. Since we have 44 sets of data in the training set (25 sets of normal data, 19 sets of abnormal data), thus, we have a label vector **r** with a dimension of 44 × 1. We also create a coefficient vector for each mode. The coefficient vector for mode *i* is represented by **b**_*i*_, whose dimension is also 44 × 1. The correlation between the mode *i* and the label is shown as

(4) correlation=|rT∗bi|rT∗r∗biT∗bi.

In this paper, we select top 4 modes from order with a large correlation value. 

**Figure 5:**
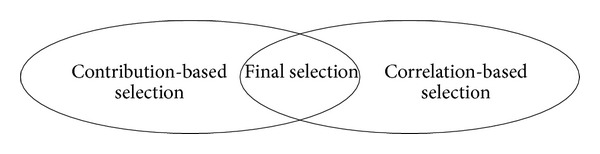
The schematic diagram of the selection of effective modes.

 Finally, we take a product set of contribution rate based selected modes and correlation based selected modes.  Figure 5 shows the schematic diagram of the proposed mode selection method.

## 4. Mapping Function Estimation 

**Figure 6:**
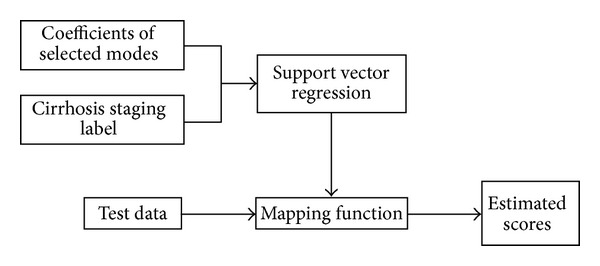
The strategy of how to estimate the stage of a new data.

 Suppose we have training data {(**b**_1_, *r*_1_), (**b**_2_, *r*_2_),…, (**b**_*N*_, *r*_*N*_)}, **b**_*i*_ is the SSM coefficient vector of the *i*th training sample, *r*_*i*_ is the stage label of the *i*th training sample, and *N* is the number of training samples. We use support vector regression [21] to estimate the stage of the cirrhotic liver. We attempt to calculate a function as (5) that can approximately map the coefficients (**b**) of selected modes to the ground truth of cirrhosis stage (*r*):

(5) r=f(b)=〈w,b〉+a,

where 〈·, ·〉 denotes the dot product, **w** and *a* are function parameters to be estimated. This mapping function will allow us to estimate the proceeding stage of a new data.  Figure 6 illustrates the proposed strategy on how to estimate the stage of the new data.

Compared to other regression strategies, Support Vector Regression (SVR) has the advantage of being usable under different kernel functions and highly accurate mapping based on parameter selection [21]. Therefore, the convex optimization problem can be given as

(6) minimize 12||w||2+C∑i=1N(ξi+ξi∗),subject  to {ri−〈w,bi〉−a≤ε+ξi〈w,bi〉+a−ri≤ε+ξi∗       ξi,ξi∗≥0,

where *C* is a positive constant and *ξ*_*i*_ and *ξ*_i_* are slack variables.

Equation (6) can be reformulated into a duel problem:

(7) maximize {−12∑i,j=1N(αi−αi∗)(αj−αj∗)k(bi,bj),−ε ∑i=1N(αi+αi∗)+∑i=1Nri(αi−αi∗),subject  to ∑i=1ℓ(αi+αi∗)=0, αi,αi∗∈[0,C],

where *α* and α* are Lagrange multipliers and *k*(**b**_*i*_, **b**) is the kernel function. The Gaussian kernel function is used in this paper for a nonlinear mapping. The obtained mapping function can be written as

(8) f(b)=∑i=1l(αi−αi∗)k(bi,b)+a.

By (8), we are able to estimate the proceeding stage of a new data with a coefficient vector of **b**.

## 5. Experimental Results

**Figure 7:**
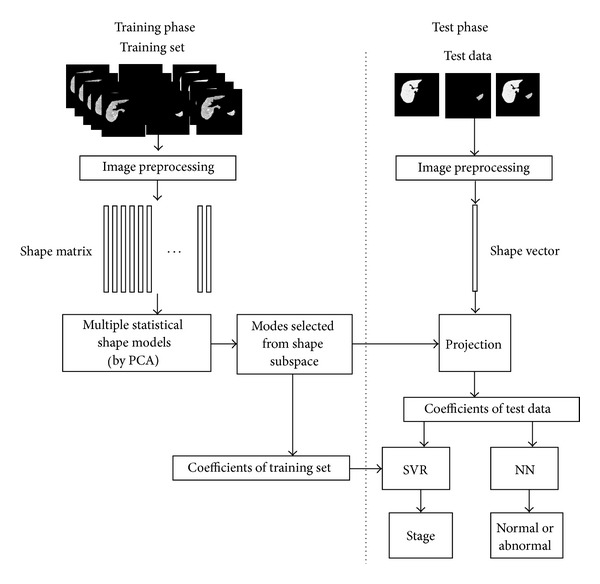
The schematic flow of our experiment.

We used 44 clinical CT datasets (25 normal data and 19 cirrhotic liver data) for this research. Among 19 cirrhotic data, 10 cirrhotic data are labeled with stages. The number and labels for each stage are shown in Table 1. We did both two-class (normal and abnormal) classification experiments and SVR based stage estimation experiments. We use all the 44 data for classification experiments and the labeled 35 data as shown in Table 1 for SVR based stage estimation experiments. We performed leave-one-out experiments to validate the effectiveness of our method. Both the two-class (normal and abnormal) classification experiment and the SVR based stage estimation experiment are done. The flow of our experiments is shown in Figure 7. 

**Figure 8:**
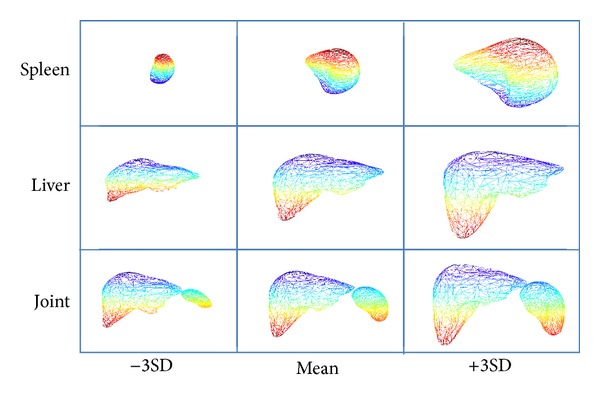
Visualized shape variations controlled by selected modes.

In the training phase, we constructed three SSMs: the liver SSM, the spleen SSM, and the joint SSM of the liver and the spleen from training data set. Based on our proposed mode selection method described in previous section, we select one mode from each SSM, respectively. Totally, three modes are selected. The shape variations of the selected modes are shown in Figure 8. The coefficients of the selected modes and the stage label are used for SVR training.

In the test phase, the test data is projected to selected modes. Their coefficients are used as features. In the two-class classification experiments, we used the simple Nearest Neighbor (NN) algorithm to classify all 44 sets of data. In the stage estimation experiments, we used SVR as a model of the mapping function as described in Section 4. In order to make a comparison, we performed both classification and stage estimation experiments with three approaches. The first approach is the same as our previous method [18, 19]. Only the liver SSM is used, and the mode (feature) selection is based on conventional AVCR. The second approach is a comparison method. The model is only the liver SSM just like our previous method (the 1st approach), but the proposed mode selection method described in Section 3 is used. The third approach is our proposed method in this paper. Multiple SSMs are used for morphological analysis with our proposed mode selection method. 

**Figure 9:**
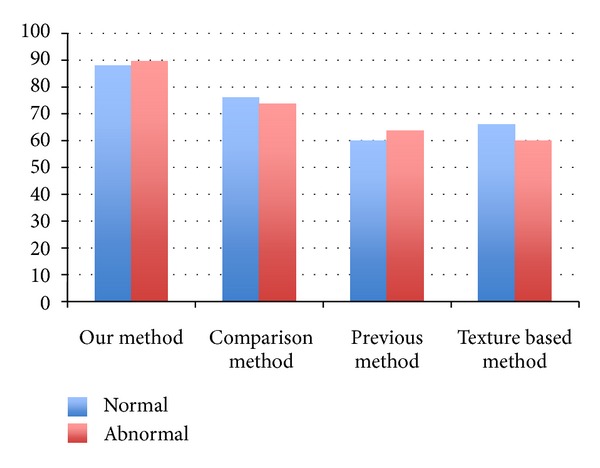
Comparison of classification accuracy among four different methods.

The comparison of classification accuracy among three approaches (our method, comparison method, and previous method) is shown in Figure 9. It can be seen that the classification accuracy for both normal livers and abnormal livers (cirrhotic livers) is significantly improved by our proposed multiple SSMs method and the mode selection method. The classification accuracies are 88% and 90% for normal and abnormal livers, respectively. 

We also compared our method with texture based method. The mean and variance of the intensity histogram are used as features of texture. The classification accuracies of the texture based method are only 66% and 60% for normal and abnormal livers, respectively, which are also shown in Figure 9.

**Figure 10:**
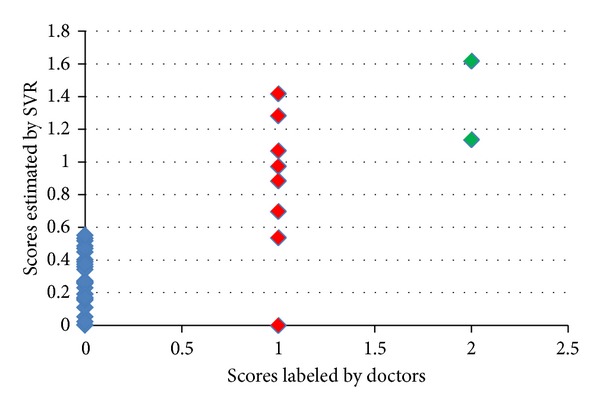
Preliminary results of cirrhosis stage estimation.

We also applied leave-one-out cross-validation for stage estimation using the labeled 35 data shown in Table 1. The preliminary results are shown in Figure 10. The horizontal axis represents the ground truth stage of tested data. The vertical axis represents the estimated scores using SVR.

As shown in Figure 10, there was one early stage data that was misclassified as normal. For the misclassified early stage case, it has very similar shape feature as normal data. Unable to handle such extreme cases is the limitation of our complete shape analysis based method. We also can notice a tiny overlap between estimated results of normal and early stage data. Besides these two defections, our method established considerably promising result on differentiating normal and cirrhotic data. 

**Figure 11:**
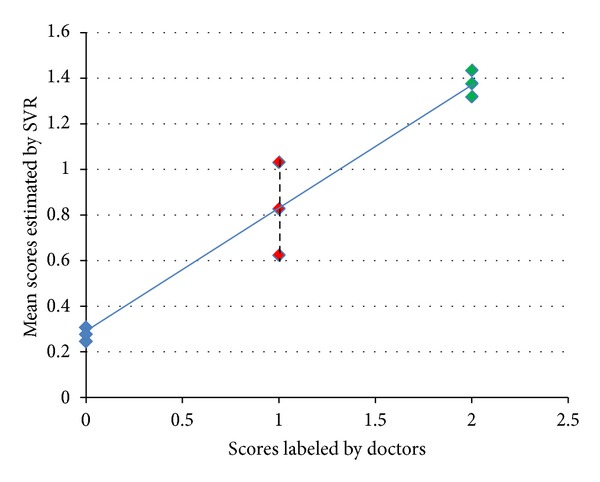
The mean score and variance of estimated results.

 Due to the data limitation, we only have two sets of middle and late stage data tested. One middle stage data can be easily distinguished from the early stage ones. The other case's score is lower than two early stage cases' scores. However, from the mean score and variance of estimated results which are shown in Figure 11, we can clearly observe an increment from normal to early stage and early stage to middle stage. This result validates the potential of our morphological analysis and machine learning based method on estimating the proceeding stage of the chronic liver diseases. 

## 6. Conclusions

We developed a multiorgan based morphological analysis method combined with a machine learning regression method to assist liver cirrhosis diagnosis and quantification. Our method can not only achieve an accurate normal/abnormal classification but also can estimate the proceeding stage of cirrhotic cases. We constructed three SSMs (the liver SSM, the spleen SSM, and the joint SSM of the liver and the spleen) for morphologic analysis of cirrhotic livers. Compared with the use of conventional single liver SSM, the classification accuracy is improved by the use of multiple SSMs for both normal and cirrhotic livers. We also proposed a mode selection method, which is based on both its accumulated variance contribution rate (AVCR) and its correlation with doctor's label scores. Compared with the conventional AVCR based method, our proposed method can also improve the classification accuracy for both normal and cirrhotic livers. The classification accuracies for normal and cirrhotic livers are 88% and 90%, respectively. We estimated the mapping function between the selected modes and the stage labels by using SVR. The proceeding stage of the cirrhotic liver can be estimated by the mapping function. Preliminary results validated the potential of our method. However, there are still several issues we want to address in the future. The first issue is to increase the number of the training samples, especially the number of middle and late stage data. The second issue is we mixed normal and abnormal data for computing the PCA. When we have more data, we will try to treat the positive and negative data separately to derive more discriminative modes. The third issue is that although we obtained considerably satisfying results, from a pathological point of view, it is highly recommended to combine both shape and texture analysis [25] to assist the diagnosis. Development of an automated method for the liver and the spleen segmentation is necessary for future automated CAD systems, and the development is under way [26]. In addition, our multiorgan-based statistical shape analysis method can be applied to assist the diagnosis of other diseases related to shape deformation. 

## Figures and Tables

**Table 1:** The labeled data used for SVR based stage estimation experiments.

	Normal	Early stage	Middle and late stages
Stage label	0	1	2
Number	25	8	2
